# Beyond the Wall: Exopolysaccharides in the Biofilm Lifestyle of Pathogenic and Beneficial Plant-Associated *Pseudomonas*

**DOI:** 10.3390/microorganisms9020445

**Published:** 2021-02-21

**Authors:** Zaira Heredia-Ponce, Antonio de Vicente, Francisco M. Cazorla, José Antonio Gutiérrez-Barranquero

**Affiliations:** 1 Departamento de Microbiología, Facultad de Ciencias, Universidad de Málaga, 29071 Málaga, Spain; zahiraheredia@uma.es (Z.H.-P.); adevicente@uma.es (A.d.V.); cazorla@uma.es (F.M.C.); 2 Departamento de Microbiología, Campus de Teatinos, Instituto de Hortofruticultura Subtropical y Mediterránea “La Mayora”, Universidad de Málaga—Consejo Superior de Investigaciones Científicas (IHSM-UMA-CSIC), 29071 Málaga, Spain

**Keywords:** exopolysaccharides, biofilm, plant-associated *Pseudomonas*

## Abstract

The formation of biofilms results from a multicellular mode of growth, in which bacteria remain enwrapped by an extracellular matrix of their own production. Many different bacteria form biofilms, but among the most studied species are those that belong to the *Pseudomonas* genus due to the metabolic versatility, ubiquity, and ecological significance of members of this group of microorganisms. Within the *Pseudomonas* genus, biofilm studies have mainly focused on the opportunistic human pathogen *Pseudomonas aeruginosa* due to its clinical importance. The extracellular matrix of *P. aeruginosa* is mainly composed of exopolysaccharides, which have been shown to be important for the biofilm architecture and pathogenic features of this bacterium. Notably, some of the exopolysaccharides recurrently used by *P. aeruginosa* during biofilm formation, such as the alginate and polysaccharide synthesis loci (Psl) polysaccharides, are also used by pathogenic and beneficial plant-associated *Pseudomonas* during their interaction with plants. Interestingly, their functions are multifaceted and seem to be highly dependent on the bacterial lifestyle and genetic context of production. This paper reviews the functions and significance of the exopolysaccharides produced by plant-associated *Pseudomonas*, particularly the alginate, Psl, and cellulose polysaccharides, focusing on their equivalents produced in *P. aeruginosa* within the context of pathogenic and beneficial interactions.

## 1. Introduction

Biofilms are matrix-enclosed bacterial populations that are adherent to each other and to surfaces and/or interfaces and are mainly composed of polysaccharides, proteins, lipids, and extracellular DNA [1,2,3]. During biofilm formation, the cells transit from a motile to a sessile lifestyle by interacting with a surface and starting to produce an extracellular matrix that holds them together and attaches them to the surface [2]. Therefore, the cells forming biofilms are referred to as sessile cells, which differ from their non-encased free-swimming counterparts, the planktonic cells [4]. Recent studies indicate that biofilms represent the main mechanism of active bacterial life due to their dominance in all habitats throughout the world [5,6]. Compared to the planktonic lifestyle, the biofilm lifestyle confers several benefits to the integrating cells, such as protection against antimicrobial agents and predators, tolerance towards changing environmental conditions, and colonization aptitudes [3,7,8].

Bacteria form biofilms in artificial and natural environments, including the soil, internal and external tissues of all living organisms, rocks, and water, among others [5]. Many different bacteria form biofilms, but the *Pseudomonas* genus is among the most studied for several reasons: (1) it harbors species with the ability to colonize a wide variety of environments due to the high metabolic and physiologic versatility found in this group of microorganisms, (2) it has ecological relevance due to its interactions with living organisms, and (3) it has potential biotechnological applications [9]. The *Pseudomonas aeruginosa* species, a ubiquitous bacterium that can also act as an opportunistic human pathogen, has long been used as a model bacterium within the *Pseudomonas* genus for the study of biofilm formation and pathogenesis due to its relevance in the clinical environment [10]. The extracellular matrix of *P. aeruginosa* has been studied in-depth and, to date, is known to contain three exopolysaccharides: alginate, polysaccharide synthesis loci (Psl), and pellicle loci (Pel) [11]. The role of these exopolysaccharides in the biofilm architecture of *P. aeruginosa* and the impact of their production in the clinical setting, such as protection against antibiotic treatments and host defenses, have been explored in several studies [12,13,14,15,16,17,18,19,20]. Although *P. aeruginosa* produces infections in humans, there are also some examples in which this bacterium can act as a pathogen for plants [21,22]. However, the biological significance of alginate, Psl, and Pel exopolysaccharides in a nonclinical context has not been studied.

Bacteria belonging to the *Pseudomonas* genus are common inhabitants of plant surfaces [23,24]. The role played by *Pseudomonas* in the agricultural industry is remarkable as several economically important activities are derived from their interaction with plants. Among these activities, there are harmful diseases that involve severe economic losses and beneficial activities such as plant growth stimulation, the promotion of plant health and nutrient availability in soils, and induction of plant immune defenses [25,26]. Pathogenic plant-associated *Pseudomonas* are predominantly present on the phyllosphere. The phyllosphere is an extreme and unstable habitat as it is exposed to highly variable nutrient and water availability, temperatures, and ultraviolet (UV) radiation. Therefore, the microbial populations associated with the phyllosphere must be adapted to these continuously fluctuating conditions [23,27,28]. The extracellular matrix of epiphytic bacteria contributes to the fitness [29,30,31], protection [8,32], and hydration of the cells [33], allowing cells to cope with these ever-changing conditions. Conversely, beneficial plant-associated *Pseudomonas* usually prevail in the rhizosphere. Compared to the phyllosphere, the environmental fluctuations that take place on the rhizosphere are weak and buffered [34]. Nevertheless, the rhizosphere is not considered a uniform and stable environment as the conditions can change abruptly in extremely short distance ranges [35,36]. Biofilm formation by beneficial plant-associated *Pseudomonas* plays advantageous roles for both the plant and bacteria [27,37]. On the one hand, they can increase plant yield by improving mineral uptake and phytohormone production, inducing the competitive suppression of pathogens and triggering plant-induced systemic resistance [38]. On the other hand, these biofilms allow the attachment of the cells to a nutrient source and confer protection against plant defenses and environmental fluctuations [27,37]. Furthermore, the biofilms produced by rhizospheric bacteria enhance soil aggregation, which improves the water-holding capacity, fertility, and porosity of the soils, leading to an increase in agricultural productivity [39,40,41,42].

Some of the biofilm components, mainly exopolysaccharides, that are required for biofilm formation and pathogenesis in *P. aeruginosa* find their equivalents in pathogenic and beneficial plant-interacting *Pseudomonas*. In this review, we shed light on the extracellular matrix exopolysaccharides of plant-associated *Pseudomonas* within the context of pathogenic and beneficial interactions.

## 2. Ecological Significance of Biofilm Formation by Plant-Interacting Bacteria

Plant-associated bacteria develop a biofilm lifestyle during their interactions with plants [27,37]. Depending on whether biofilms are formed by pathogenic or beneficial individuals, the ecological outcome resulting from the interaction can be completely different. In the context of pathogenic plant-associated bacteria, the role of different components involved in biofilm formation has been studied. For instance, the biofilms formed by *Erwinia amylovora*, the causal agent of fire blight disease in different plant species of the *Rosaceae* family, and specifically the amylovoran and levan exopolysaccharides, physically blocked the vascular system of plants [43,44,45]. A mutant of *Ralstonia solanacearum*, the causal agent of bacterial wilt disease, in the *lecM* gene, which encodes a lectin, showed reduced biofilm formation in vitro and colonization of the intercellular spaces of tomato leaves and was impaired in virulence [46]. The *gumB* mutant of *Xanthomonas citri*, which produces canker disease in citrus plants, was unable to produce the polysaccharide xantan and exhibited reduced biofilm formation, survival and symptom development on lemon leaves [30]. Similarly, *Xylella fastidiosa*, which causes economically important diseases in several host plants, produced exopolysaccharides that played roles in the virulence of this bacterium, as these are required for bacterial movement within plants and plant-to plant transmission through insects [47,48].

Notably, the *Pseudomonas syringae* complex harbors most of the phytopathogens within the *Pseudomonas* genus [49,50]. In particular, the species *P. syringae* is one of the most ubiquitous bacterial participants of the phyllosphere [51]. This ubiquity, together with the fact that it can infect almost all important agricultural crops [25,52], has made it a model for the study of plant–bacteria interactions. *P. syringae* possesses a great diversity of virulence factors that engage in plant infection, as well as adaptation mechanisms that improve bacterial survival over the plant surface. Generally, *P. syringae* produces a type III secretion system (T3SS), effector proteins, motility appendages, phytotoxins, multidrug efflux pumps, extracellular polysaccharides, cell wall-degrading enzymes, and ice nucleation activity [53]. Copper- and UV radiation-resistance genes, as well as exopolysaccharide production, play fundamental roles in *P. syringae* fitness and survival [31,50,54,55,56,57,58]. In *P. syringae* pv. syringae (Pss), biofilm formation has been proven to influence the transition between pathogenic and epiphytic lifestyles in plants [29,31,59].

In the context of beneficial plant-associated bacteria, *Bacillus subtilis*, a Gram-positive bacterium that acts as a biocontrol agent of several plant pathogens, requires the production of extracellular matrix components involved in biofilm formation, such as those encoded by *tapA-sipW-tasA* and *epsA-O* operons, for the colonization of the plant roots and for conferring plant protection [60]. *Pseudomonas fluorescens*, an important rhizobacterium that promotes plant health and nutrition, requires biofilm formation for the colonization of plant surfaces [61]. A cellulose exopolysaccharide mutant in the *P. fluorescens* SBW25 strain was compromised in the colonization of the rhizosphere and the phyllosphere of sugar beet compared to the wild-type strain [61]. In general, the *P. fluorescens* species and some closely related species that belong to the *P. fluorescens* complex are among the most studied bacteria within soil communities, because they frequently show agricultural, biotechnological, and ecological interest, mostly due to their beneficial plant features [62]. In particular, the *P. fluorescens* and *Pseudomonas chlororaphis* species stand out because of their potential use as biocontrol agents as they frequently contribute to plant health by exerting antagonist activities against pathogens [63,64,65]. Phenotypes linked to biofilm formation have also been observed to favor bacteria–plant root interactions and biocontrol activity of *P. chlororaphis* and *Pseudomonas putida* species [66,67,68,69,70]. Usually, biocontrol agents can form biofilms, and increasing evidence strongly suggests that biofilm-forming ability should be considered in assessing their potential beneficial performance [71].

## 3. Main Exopolysaccharides Produced by Plant-Associated *Pseudomonas*

Among all the exopolysaccharides that are produced by plant-associated *Pseudomonas* [72], those that have been mainly studied are alginate, cellulose, and Psl (Table 1). A description of their functions in biofilm formation and architecture and their ecological significance during pathogenic and beneficial plant–bacteria interactions are listed below.

### 3.1. Alginate Exopolysaccharide

Alginate is a copolymer made of O-acetylated D-mannuronic and L-glucuronic acid residues joined by β-1,4 linkages [74]. In PAO1, the alginate polysaccharide is encoded on a twelve gene operon that corresponds to the PA3540-PA3551 genomic region [13]. During infections in cystic fibrosis (CF) patients, *P. aeruginosa* undergoes a switch into a mucoid phenotype characterized by alginate overproduction [75,76,77]. Alginate overexpression increases the resistance of *P. aeruginosa* to antimicrobial treatments, predators, and host defenses [12,78]. The high frequency in which this conversion occurs, and the protective capacities described for alginate, suggests that alginate is the main exopolysaccharide of the *P. aeruginosa* extracellular matrix. However, studies performed on nonmucoid *P. aeruginosa* strains (e.g., PAO1 and PA14), the truly predominant phenotype and the one responsible for the colonization of the lungs of CF patients [13], have shown that, although it is not critical for biofilm constitution, this polysaccharide is a component of the *P. aeruginosa* extracellular matrix and can influence its biofilm architecture [13,14,79,80].

Studies performed on alginate in some plant-associated *Pseudomonas* have revealed that this polysaccharide plays minor structural roles in their biofilms, including the bacterial phytopathogen *P. syringae* and the plant-beneficial bacteria *P. fluorescens*, *P. chlororaphis,* and *P. putida* [59,70,73,81,82]. The alginate-deficient derivative of the *P. syringae* pv. glycinea PG4180.muc strain formed biofilms to the same extent as the wild-type strain in flow-cell chambers [81]. However, the biofilm architecture of the PssUMAF0158 *∆alg8* strain, which does not produce alginate, showed slightly but significantly lower surface coverage and volume than the wild-type strain [59], as was previously described in *P. aeruginosa* [80]. Alginate is overproduced in some strains of *P. syringae* upon exposure to copper bactericides, which are usually applied to reduce the disease incidence caused by some plant pathogens [32]. This could be explained because exopolysaccharide production has been generally associated with a higher tolerance against toxic compounds [2,83]. Previous works have indicated that alginate polysaccharides are involved in the pathogenic interaction of *P. syringae* with plants [29,84,85]. For instance, the alginate mutant of the *P. syringae* pv. syringae 3525 strain, the causal agent of bacterial brown spot on bean, is significantly impaired in the colonization of bean (host) and tomato (non-host) leaves, and although it retains the ability to generate symptoms, the symptoms are less severe than those induced by the wild-type [29]. However, these results have not been observed in other *P. syringae* strains [59,86,87,88]. For example, in *P. syringae* pv. glycinea PG4180 strain, the causal agent of bacterial blight of soybean, the expression of the AlgT regulator protein, but not alginate production *per se*, promotes survival and symptom development in plants [88]. Similarly, the PssUMAF0158 *∆alg8* mutant strain is not altered in the induction of symptoms in tomato compared to the wild-type strain [59].

The structural functions displayed by alginate in the biofilms of plant-pathogenic *Pseudomonas* are in line with those observed in the plant-beneficial *Pseudomonas*. The alginate mutant of *P. fluorescens* SBW25 still forms biofilms in flow-cell chamber experiments, but they are thinner than those formed by the wild-type strain [82]. This result is consistent with the flow-cell chamber phenotypes of the PssUMAF0158 and PAO1 alginate mutant derivative strains [59,80]. However, the alginate mutant of the biocontrol agent *P. chlororaphis* PCL1606 (PcPCL1606 ∆*alg8*) forms biofilms to the same extent as the wild-type in flow-cell chamber experiments and is not impaired in initial surface attachment, showing nonsignificant differences in surface coverage and volume values with respect to the wild-type [70]. Alginate has been described as the primary polysaccharide that promotes hydration under desiccating stress in *P. putida* [89,90]. In fact, alginate slightly contributes to the biofilm architecture of *P. putida* under water-limiting conditions [90]. The functions performed by alginate polysaccharide in both *P. fluorescens* and *P. putida* strains in vivo seem to be more relevant than those in vitro. For instance, the CHA211 and CHA213M mucoid variants of the *P. fluorescens* CHA0 strain, which overproduce alginate, enhance their biofilm formation abilities on carrot roots compared to the wild-type strain [91]. The genomic region located upstream of the *algD* gene of *P. putida* KT2440 is active during the colonization of maize root, which suggests that this polysaccharide could be a fitness determinant for the rhizosphere colonization ability of this bacterium [92]. Overall, these studies indicate that alginate is not a critical component for biofilm formation in vitro in plant-associated *Pseudomonas* and that its role seems to be more prominent in vivo, facilitating colonization and providing protection against stressors.

### 3.2. Cellulose Exopolysaccharide

Cellulose is a polymer made of D-glucose residues joined by β-1,4 glycosidic linkages and is considered a relevant biofilm matrix molecule in many environmental *Pseudomonas* species [93,94]. Several biosynthesis and regulation mechanisms have been described for bacterial cellulose, but a common role of this component is to facilitate the establishment of efficient host-bacteria interactions [95]. Previous studies reported that several plant-associated *Pseudomonas* species can produce cellulose, including the plant-associated pathogenic bacteria *P. syringae*, *P. asplenii*, *P. marginalis*, *P. corrugate,* and *P. savastanoi* and beneficial bacteria, such as *P. fluorescens* and *P. putida* [72,93,94]. Within the *Pseudomonas* genus, *P. fluorescens* SBW25 (SBW25) is traditionally used as the model strain for the study of bacterial cellulose. In SBW25, cellulose polysaccharide is encoded on a ten-gene operon (*wssA-J*) that corresponds to the PFLU0300-PFLU0309 genomic region [96]. This exopolysaccharide is involved in the formation of floating biofilms, also called pellicles, in many strains of the species mentioned above, including the SBW25 [31,93,96,97,98,99]. The *P. aeruginosa* species does not contain the cellulose operon [72]. In particular, *P. aeruginosa* PAO1 and PA14 strains, which have been traditionally used as model strains for conducting biofilm studies within the *Pseudomonas* genus, were tested for cellulose production, but in line with in silico observations [72], they were not found to produce this exopolysaccharide [94]. However, the PAO1 and PA14 strains contain a seven-gene operon that encodes Pel, which is a polymer composed of partially acetylated 1→4 glycosidic linkages of N-acetylgalactosamine and N-acetylglucosamine [100]. The *pel* operon is poorly conserved among environmental *Pseudomonas* [72,100,101]. Interestingly, Pel promotes the formation of pellicle biofilms, as has also been described for cellulose [102].

Among all plant-pathogenic *Pseudomonas* that have been reported to produce cellulose, studies regarding its structural roles within biofilms and biological significance have essentially been conducted on *P. syringae*. The *P. syringae* pv. syringae (Pss) UMAF0158 (PssUMAF0158) strain, the causal agent of bacterial apical necrosis (BAN) on mango trees, and *P. syringae* pv. tomato DC3000 (PtoDC3000), responsible for bacterial speck disease on tomato plants, produce cellulose as the main exopolysaccharide of their biofilms [31,59,103]. The biofilm structures formed by PssUMAF0158 and PtoDC3000 in micro-well plates are highly similar, consisting of pellicles with wrinkles on the surface that are weakly attached to the walls of the culture vessels [31,103]. Despite the structural similarities found in vitro, the biological performance of cellulose seems to differ in both strains. Cellulose allows PssUMAF0158 to adhere to mango leaves, and its production intimately affects the epiphytic and pathogenic stages of this strain over the plant surface [31]. Hence, the incidence and severity of necrotic symptoms developed by PssUMAF0158 on tomato leaflets are lower in the wild-type than in cellulose mutants (∆*wssB* and ∆*wssE* mutants) and practically nonexistent in the cellulose-overproducing strain [31]. These results, together with the fact that the highest BAN symptoms coincide with cool and wet periods [104], support the proposed lifecycle of Pss strains over the mango tree, in which biofilm formation would be mainly needed during the epiphytic phase (spring/summer) when the bacteria are more exposed to the external environment, and protection against its challenging conditions becomes crucial for survival [50]. Interestingly, the link observed between cellulose production and PssUMAF0158 transition through epiphytic and pathogenic stages over the mango plant surface has not been reported in PtoDC3000. The disease symptoms developed in tomato by the PtoDC3000 wild-type strain were not different from those of its *∆wssBC*-derived mutant [105]. Furthermore, in disagreement with what has been observed in PssUMAF0158, cellulose overproduction in PtoDC3000 does not lead to a significant impact on virulence [103]. However, the PtoDC3000 *armZ* gene mutant, which does not produce alginate and does overproduce cellulose, has a reduced virulence compared to the wild-type strain [105]. Although PssUMAF0158 and PtoDC3000 are categorized as *P. syringae* species and belong to the *P. syringae* complex, this complex is comprised of a hodgepodge that, in effect, includes many other taxonomically related species [49]. A previous study revealed that the phylogenetic relationship between *P. syringae* pv. syringae B728a strain, closely related to PssUMAF0158, and PtoDC3000, is not very proximate. In fact, PtoDC3000, together with other strains of the tomato pathovar, seems to form a new species *Pseudomonas tomato*, pending a deeper taxonomic analysis [49]. This evidence, together with the fact that the infection assays were performed using different tomato cultivars and inoculation approaches, could all eventually account for different results.

Regarding beneficial plant-interacting *Pseudomonas*, studies on bacterial cellulose have been mainly conducted on *P. fluorescens* and *P. putida* species. Biofilm experiments on SBW25 determined that the gradients occurring within a static microcosm immediately select for the emergence of variants that occupy different niches [106]. Among those variants, the air-liquid (A–L) interface is colonized by wrinkly spreader (WS) pellicles, an SBW25-derived mutant that overproduces cellulose compared to the wild-type equivalent [107]. In *P. putida* mt2 and its plasmid-free derivative KT2440 [108] strains, cellulose plays minor roles in biofilm formation in vitro [73,89], while two additional exopolysaccharide gene clusters, putida exopolysaccharide A (Pea) and putida exopolysaccharide B (Peb), are essential for biofilm formation in this species [73,89]. Instead, the role of cellulose exopolysaccharide in *P. putida* seems to be directed more towards conferring protection, as water-limiting conditions and increasing osmolarity highly induce cellulose expression of *P. putida* mt2 [89,109]. In addition, the cellulose mutant of *P. putida* mt2 strain accumulates significantly more reactive oxygen species (ROS) than the wild-type strain upon exposure to matric and copper stressors [109]. During plant–bacteria interactions, the cellulose exopolysaccharide of SBW25 contributes to the ecological performance of this strain in the rhizosphere and phyllosphere of sugar beet [61]. Thus, a cellulose-defective mutant of SBW25 (SM13) was compared against the wild-type in the rhizosphere, phyllosphere, and bulk soil surrounding the rhizosphere of sugar beet seedlings, and the results showed no significant differences between the fitness of SM13 relative to the wild-type in bulk soil, but significant differences were found in the rhizosphere and phyllosphere, especially in the phyllosphere [61]. Something similar has been reported in the *P. putida* mt2 strain in which the cellulose mutant is impaired in the colonization of the maize rhizosphere during competition with the wild-type equivalent [73]. These studies indicate that, while the cellulose operon does not seem to be critical for biofilm formation under laboratory conditions in *P. fluorescens* and *P. putida*, their roles in these species seem to be more pronounced in vivo.

### 3.3. Psl Exopolysaccharide

The Psl polysaccharide was first described in *P. aeruginosa* [102,110,111], and its structural analysis determined that it consists of a repeating pentasaccharide subunit of D-mannose, D-glucose, and L-rhamnose in a 3:1:1 ratio [112]. In PAO1, Psl was formerly described to be encoded by the 15-gene operon *psl* (*pslA-O*), which corresponds to the PA2231-PA2245 genomic region [102,110,111]. However, later works revealed that the last three genes of the operon (*pslMNO*) constitute an independent transcriptional unit [113,114,115] and are not truly required to produce Psl [112]. Except for the case of the *P. aeruginosa* PA14 strain, which does not produce Psl due to the absence of *pslA-D* genes [102], the *psl* gene cluster is present in multiple strains of *P. aeruginosa* [72,80], where it plays key roles in their biofilm lifestyles [80]. Several studies have proven the involvement of Psl in adhesion to biotic and abiotic surfaces, biofilm architecture, motility, and protection against stressors [16,19,116,117,118,119]. Although research on Psl polysaccharides has been mostly conducted in *P. aeruginosa*, the existence of a *psl*-like gene cluster has been reported in some environmental nonaeruginosa *Pseudomonas* [59,70,72,101]. Generally, the *psl*-like gene clusters found in nonaeruginosa *Pseudomonas* either lack orthologues to *pslMNO* genes or are found scattered in the genome outside the cluster. The bacterial phytopathogen PssUMAF0158 contains a *psl*-like gene cluster that does not include orthologues to the *pslCLMNO* genes and encodes a putative acetyltransferase between the *pslJ*-and *pslK*-like genes that might perform a related function to that of acyltransferase PslL [59]. Interestingly, the *psl*-like gene cluster of PssUMAF0158 seems to be highly conserved among the plant-associated phylogroups belonging to the *P. syringae* complex [59]. The biocontrol agent PcPCL1606 also contains a *psl*-like gene cluster, which lacks the *pslLMNO* genes and encodes a putative acetyltransferase between the *pslJ*- and *pslK*-like genes, similar to that of PssUMAF0158 [70]. However, the *psl*-like gene cluster of PcPCL1606 is not present in some phylogroups of the *P. fluorescens* complex and is partially present in others, according to the strains included in a previous study [70]. It is completely absent in the *corrugata*, *jessenii*, and *koreensis* phylogroups; only present in *Pseudomonas* GM21 strain of the *mandelii* phylogroup; and is partially present within the *P. fluorescens* phylogroup. Interestingly, a *psl*-like gene cluster is found in all the strains of the *P. chlororaphis* phylogroup that have been assessed [70], which suggests that this polysaccharide could be relevant for biofilm formation in this species.

The first study regarding Psl composition in *P. aeruginosa* PAO1 determined that this polysaccharide was a galactose- and mannose-rich exopolysaccharide [120]. Support for this information came from three pieces of evidence. First, a chemical composition analysis of exopolysaccharide preparations of WFPA801, a PAO1-derived Psl-inducible strain, determined the presence of galactose, mannose, and glucose, as well as trace amounts of xylose, rhamnose, and N-acetylglucosamine. Second, staining of planktonically grown WFPA801 cells with FITC-HHA lectin, which binds to some mannosyl units, and FITC-MOA lectin, which binds to some galactosyl units, revealed green fluorescent signals on the WFPA801 surface. Ultimately, mutants of the *pslH* gene, which encodes a putative galactosyltransferase, and the *pslI* gene, which encodes a putative mannosyltransferase, were deficient in attachment, yielding a similar phenotype to that of the WFPA800 null Psl-producing strain [120]. Two years later, the structural analysis of Psl was published, indicating that it likely consisted of a pentasaccharide repeating unit of mannose, glucose, and rhamnose in approximate ratios of 3:1:1 [112]. Interestingly, galactose, which was reported as the major component of Psl in the first study [120], was not detected as a component of Psl in the structural analysis [112]. The authors stated that different growth conditions were used in both studies, which could account for some variations in composition, as described previously [121]. Therefore, there is some thought that different forms of Psl might be produced even in the same strain depending on the growth conditions. Be that as it may, mannose seems to be a key component of the Psl structure in *P. aeruginosa*. An analysis of the composition and structure of the putative Psl polysaccharide produced by PssUMAF0158 and PcPCL1606 has not yet been conducted, but some hints exist regarding the existence of a polysaccharide that resembles Psl in *P. syringae* and *P. fluorescens*. Thus, it was reported that, in addition to alginate and levan, *P. syringae* PG4180 produced a third exopolysaccharide (EPS) that consisted of a fibrous structure in its biofilms and bound to *Naja mossambica* lectin (NML) [81]. Interestingly, the monosaccharide specificity of NML is mannose [122]. Furthermore, two *P. fluorescens* strains isolated from rotted bell pepper, PF-05-2 and PM-LB-1, produced a novel exopolysaccharide composed of mannose, rhamnose, and glucose (1:1:1 molar ratio) substituted with pyruvate and acetate [123]. The biofilm formed by the PcPCL1606 wild-type strain but not its Psl-like-derived mutant, contains a polymer that binds to banana lectin, which also binds to mannose residues [70,124].

The ∆*pslAB* mutant of PAO1 is severely attenuated in biofilm initiation and biofilm development in flow-cell chamber experiments [110,111]. Interestingly, similar results were observed in the biocontrol strain PcPCL1606, in which the ∆*pslE* mutant was severely affected in early surface attachment and development of a mature biofilm architecture compared to the wild-type strain [70]. The biofilms developed by PAO1 and PcPCL1606 wild-type strains showed an intricate architecture in flow-cell chambers, which consisted of a multilayer of cells that covered the chamber surfaces [70,110,111]. However, the biofilm phenotype of the PAO1 ∆*pslAB* and PcPCL1606 ∆*pslE* mutant strains consisted of a monolayer of loosely aggregated cells, which suggests that this exopolysaccharide could also be important for cell-to-cell interactions [70,110,111]. Similarly, the Psl-like polysaccharide of the phytopathogen PssUMAF0158 is also involved in biofilm architecture [59]. Compared to the more developed biofilm of wild-type PssUMAF0158, the PssUMAF0158 ∆*pslE* biofilms consisted of scattered cell aggregates across the flow-cell chamber surface [59]. These cell aggregates were disrupted in the double mutant ∆*wssE,pslE* strain, which did not produce both cellulose and Psl-like polysaccharides [59]. Curiously, this phenotype was also observed in some *P. aeruginosa* strains, where the cell aggregates formed by their derived ∆*psl* mutants were disrupted in the ∆*psl*∆*pel* double mutants, affected in both Psl and Pel polysaccharide production [116]. The fact that cellulose and Pel polysaccharides are both involved in the formation of pellicle biofilms [101], and that the cell aggregates formed by these *Pseudomonas* ∆*psl* strains are disrupted when either cellulose or Pel is not produced, indicates that both polysaccharides could play redundant structural roles within biofilms, as has been previously suggested [59,101]. Indeed, it is not common to find both genomic regions encoding cellulose and Pel in the same *Pseudomonas* strain. Thus, just 12 out of 600 *Pseudomonas* genomes (2%) that have been analyzed in a recent study [72]—which belong to four different groups: *P. asplenii*, *P. fluorescens*, *P. fragi* and *P. oryzihabitans*—possess both clusters (Table 2), although whether they are functional remains unknown. With these recent data, the identity and coverage of both clusters have been analyzed in these 12 *Pseudomonas* spp. strains using the *wss* operon of SBW25 and *pel* operon of PAO1 as references.

Swarming motility and biosurfactant synthesis are coordinated with Psl production in *P. aeruginosa*, as the PAO1 Psl-deficient strain exhibited a hyperswarming phenotype due to an increase in rhamnolipid production, and vice versa [118]. Curiously, the link found between biofilm formation, rhamnolipid production, and motility in the bacterial phytopathogen PssUMAF0158 seems opposite to that described in *P. aeruginosa*. Therefore, the PssUMAF0158 ∆*pslE* mutant is impaired in swarming motility compared to the wild-type strain, and this impairment could be due to a reduction in surfactant production, as the *rhlA* gene involved in rhamnolipid precursor synthesis is downregulated in the mutant compared to the wild-type strain [59].

The PAO1 ∆*pslAB* mutant is deficient in biofilm initiation due to its reduced ability to interact with biotic and abiotic surfaces [16,110,111]. In line with these data, the recently described Psl-like exopolysaccharides of the bacterial phytopathogen PssUMAF0158 and biocontrol agent PcPCL1606 have also been reported to be involved in early surface interactions. The PssUMAF0158 ∆*pslE* mutant is impaired in early adhesion to mango leaves [59] and the PcPCL1606 *∆pslE* mutant was impaired in early surface attachment to polystyrene micro-well plates and avocado root surfaces [70]. Moreover, biofilm formation by PssUMAF0158 and PcPCL1606 strains through Psl-like exopolysaccharide biosynthesis also contributes to the lifestyles displayed by these bacteria during interaction with their plant host [59,70]. The inability to produce some extracellular matrix components, such as cellulose and Psl-like exopolysaccharides, seems to predispose PssUMAF0158 to the pathogenic lifestyle, as the mutants impaired in the production of these exopolysaccharides are significantly more virulent than the wild-type [31,59]. Consequently, the biofilm lifestyle of PssUMAF0158 could predominate during the epiphytic phase, as has been previously suggested [50]. Furthermore, the Psl-like exopolysaccharide of PcPCL1606 contributes to the biocontrol activity of this bacterium against white rot root disease caused by *Rosellinia necatrix* in avocado plants [70]. Thus, PcPCL1606 ∆*pslE* is severely compromised in disease suppression, probably because early attachment and biofilm impairments could lead to inefficient colonization of roots, which is a prerequisite for efficient disease control [70,125,126,127]. The presence of a *psl*-like cluster in some environmental pseudomonads with different lifestyles suggests that this polysaccharide might constitute a general feature of biofilm formation of these bacteria, providing different functions depending on the genetic context and niche of production.

## 4. Brief Summary and Future Perspectives

**Figure 1:**
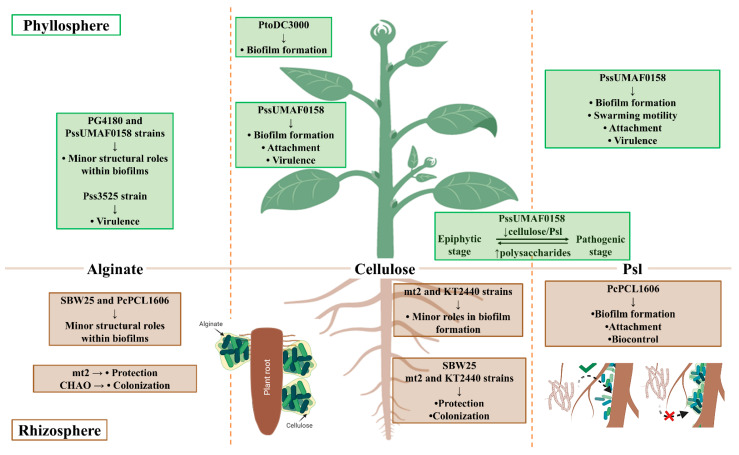
Summary of functions described for exopolysaccharides produced by plant-associated *Pseudomonas*. *P. syringae* pv. syringae UMAF0158 (PssUMAF0158), *P. syringae* pv. syringae 3525 (Pss3525), *P. syringae* pv. tomato DC3000 (PtoDC3000), *P. syringae* pv. glycinea PG4180 (PG4180), *P. fluorescens* SBW25 (SBW25), *P. putida* mt2 (mt2), *P. putida* KT2440 (KT2440), *P. fluorescens* CHAO (CHAO), and *P. chlororaphis* PCL1606 (PcPCL1606). Image created with Biorender.com.

The *Pseudomonas* genus includes species with high metabolic and physiologic versatility, as well as broad potential for adaptation to fluctuating environmental conditions, which accounts for their ability to colonize such a wide variety of environments [9]. Within the *Pseudomonas* genus, the *P. aeruginosa* species has been traditionally used as a model bacterium for the study of biofilm formation due to its impact in clinical settings [10]. The extracellular matrix of *P. aeruginosa* is predominantly composed of polysaccharides [11], and interestingly, some of them are also produced by several pathogenic and beneficial plant-associated *Pseudomonas* [72]. These exopolysaccharides display structural and/or protective roles in plant-associated bacteria. The ecological significance derived from their production is dependent on the lifestyles displayed by these bacteria during plant–bacteria interactions (Figure 1). To date, alginate polysaccharides seem to play minor structural roles in the biofilms of plant-associated *Pseudomonas* in vitro, which correlates with previous results observed in *P. aeruginosa* [13,59,70,80,82]. However, its functions that have been described in vivo, together with those directed towards protection against external stressors, such as desiccation, seem more prominent [12,78,89,90,128]. Cellulose is not produced by the *P. aeruginosa* PAO1 and PA14 model strains, but is produced by several plant-associated bacteria, including *P. syringae* and *P. fluorescens*, frequently constituting the main architectural component of their biofilms [93,94]. Instead, PAO1 and PA14 strains produce Pel, which is poorly conserved among environmental nonaeruginosa *Pseudomonas* and is responsible for pellicle formation, as previously described for cellulose [101,102]. Overall, cellulose is described as a major component of the biofilm architecture produced by several plant-associated *Pseudomonas* [31,96,105]. The Psl polysaccharide, which was first described in *P. aeruginosa*, is a key component of the biofilm architecture of this bacterium [102,110,111]. For many years, the role of Psl in biofilm formation by some environmental nonaeruginosa pseudomonads was unknown. However, the involvement of a Psl-like polysaccharide in the biofilm architecture and lifestyles of two plant-associated *Pseudomonas* species has been recently described for the first time [59,70].

Despite all the knowledge developed from biofilm studies, numerous aspects remain underexplored, for example, how different components interact within the extracellular matrix. Lectin staining has allowed detection of some polysaccharides, such as Psl and Pel polysaccharides, within the biofilms of *P. aeruginosa* [100,129], as well as alginate and levan within the biofilms of *P. syringae* [81], but how they are located with respect to one another has not been specified. Similarly, whether Psl and cellulose can interact in the extracellular matrix of the PssUMAF0158 strain remains unknown. The involvement of Psl in biofilm formation by environmental *Pseudomonas* has been overlooked for a long time, and its revealed importance in the biofilm architecture and influence on the bacterial lifestyle of the phytopathogen PssUMAF0158 and biocontrol agent PcPCL1606 could lead future studies towards determining the functions and capacities of this component in other bacterial species. Furthermore, future studies should also contemplate the compositional and structural analysis of the Psl-like polysaccharide produced by environmental pseudomonads to determine its level of resemblance to the archetypal *P. aeruginosa*.

On another note, it is currently known that specific climate factors, such as temperature, pH, light, and humidity, influence biofilm formation [130,131,132,133]. However, little information exists regarding the direct impact that climate conditions can have on plant–bacteria interactions through biofilm formation or biofilm-related processes. For example, it has been described that white light exposure, specifically blue light, increases the attachment of PtoDC3000 to *Arabidopsis thaliana* leaves [134]. Recently, the impact of temperature on the biofilm architecture of *P. aeruginosa*, guided by exopolysaccharide synthesis, has been revised [135]. More studies should focus on the direct impact that specific and combined different climate factors have on biofilm formation, particularly the impact derived from such changes on the bacterial ecology during plant–bacteria associations.

Finally, studies on extracellular polysaccharides produced in monospecies biofilms have provided interesting information regarding their roles in biofilm architecture, as well as their influence on host–bacteria interactions, but future works should be more directed towards polymicrobial biofilms. This is because environmental habitats, such as those encountered on plant surfaces, are known to harbor complex microbial assemblages [136,137,138], in which usually different species, and even different kingdoms, interact. Currently, more details regarding multispecies biofilms are being revealed [139,140,141], but there is still much work to do regarding this issue.

## Figures and Tables

**Table 1:** Main exopolysaccharides produced by different *Pseudomonas* spp. strains that are involved in biofilm formation.

Strain	Clusters Encoding the Main Exopolysaccharides Described in *Pseudomonas* ^1^			
	*alg*	*wss*	*psl*	*pel*
*P. aeruginosa* PAO1	+ ^2^	- ^2^	+	+
*P. aeruginosa* PA14	+	-	-	+
*P. syringae* pv. syringae B728a	+	-	+	-
*P. syringae* pv. tomato DC3000	+	+	+	-
*P. savastanoi* pv. phaseolicola 1448A	+	+	+	-
*P. syringae* pv. syringae UMAF0158	+	+	+	-
*P. fluorescens* SBW25	+	+	+	-
*P. fluorescens* Pf0-1	+	-	-	-
*P. fluorescens* F113	+	-	-	-
*P. chlororaphis* PCL1606	+	-	+	-
*P. chlororaphis* O6	+	-	+	-
*P. chlororaphis* subsp. aureofaciens 30–84	+	-	+	-
*P. putida* KT2440	+	- ^3^	-	-

^1^ The *wss* operon (cellulose) of *Pseudomonas fluorescens* SBW25 and the alginate (*alg)*, *psl* and *pel* operons of *Pseudomonas aeruginosa* PAO1 strains were used to perform BLASTN discontiguous megablast searches against the genome of several strains belonging to different *Pseudomonas* species. ^2^ +, presence of the exopolysaccharide gene cluster; -, absence of the exopolysaccharide gene cluster. ^3^ The *wss* cluster of *Pseudomonas putida* KT2440 strain (PP2629-PP2638 genomic region) was not detected using the *wss* operon of the *P. fluorescens* SBW25 strain. However, a *wss* cluster has been previously reported to be present in this strain [73].

**Table 2:** *Pseudomonas* spp. strains obtained from Blanco–Romero et al., (2020) that have been reported to contain the *wss* and *pel* clusters.

Strain	*wss* Cluster ^1^		*pel* Cluster ^1^	
	Identity (%)	Coverage (%)	Identity (%)	Coverage (%)
*P. agarici* NCPPB 2472	71.03	82	69.68	93
*P. azotoformans* F77	82.58	100	70.63	89
*P. azotoformans* LMG_21611	83.72	99	70.50	91
*P. extremorientalis* LMG 19695	89.82	99	70.59	92
*P. lundensis* AU1044	71.31	11^2^	72.32	91
*P. lurida* L228	83.17	100	71.37	85
*P. lurida* MYb11	82.99	100	71.48	85
*P. oryzihabitans* USDA-ARS-USMARC-56511	68.70	53	71.33	98
*P. oryzihabitans* FDAARGOS_657	70.45	57	71.50	98
*P. psychrotolerans* PRS08-11306	70.40	58	71.66	98
*P. psychrotolerans* CS51	70.11	50	74.27	94
*P. trivialis* IHBB745	91.38	99	73.28	91

^1^ The *wss* operon (cellulose) of *P. fluorescens* SBW25 and the *pel* operon of *P. aeruginosa* PAO1 were used to perform BLASTN discontiguous megablast searches against the genome of the 12 *Pseudomonas* strains that harbor both exopolysaccharide gene clusters. ^2^ The low coverage obtained in *P. lundensis* AU1044 with regard to the SBW25 cellulose cluster could be due to different approaches performed to assess the presence of exopolysaccharide clusters.
